# Ultrasound Evaluation of Congenital Cervical Teratoma and Therapeutic Management (Ex Utero Intrapartum Treatment)

**DOI:** 10.1155/2012/597489

**Published:** 2012-12-19

**Authors:** Pablo Padilla Iserte, Amparo Sanroma Pérez, Blanca Ferri Folch, Juan Rubio Moll, Vicente Diago Almela, Alfredo Perales-Marín

**Affiliations:** Department of Obstetrics and Gynaecology, University Hospital La Fe, Bulevar Sur s/n, 46026 Valencia, Spain

## Abstract

The ultrasound evaluation of the fetal neck has a high importance as a key point of the airway and digestive tract. We report the case of a fetus diagnosed with a cervical teratoma by ultrasound, which generated a compressive effect on airway, requiring a surgical approach EXIT (ex utero intrapartum treatment) to ensure the extrauterine viability.

## 1. Introduction

The ultrasound evaluation of the fetal neck is increasingly important, because it has a high impact on fetal adaptation to extrauterine life. The presence of masses in this location can cause obstructive problems at airway, producing complications during the course of pregnancy, as the development of polyhydramnios. But the biggest problem is after delivery, with the appearance of acute neonatal asphyxia with newborn death, if you do not carry out invasive techniques for resolution.

One of these approaches is the realization of EXIT surgery, a technique designed to allow partial fetal delivery by cesarean section, with establishment of a secure airway, using intubation, bronchoscopy, or tracheostomy, while fetal oxygenation is maintained by the uteroplacental circulation.

## 2. Case Presentation

**Figure 1:**
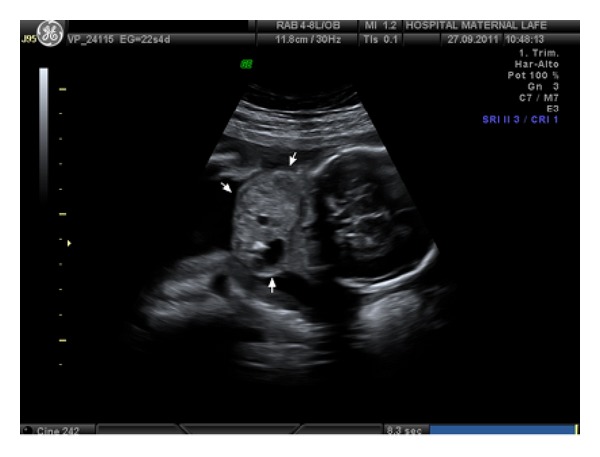
Ultrasound at 22 weeks, where heterogeneous tumor is found in cervical position, moderately vascularized.

**Figure 2:**
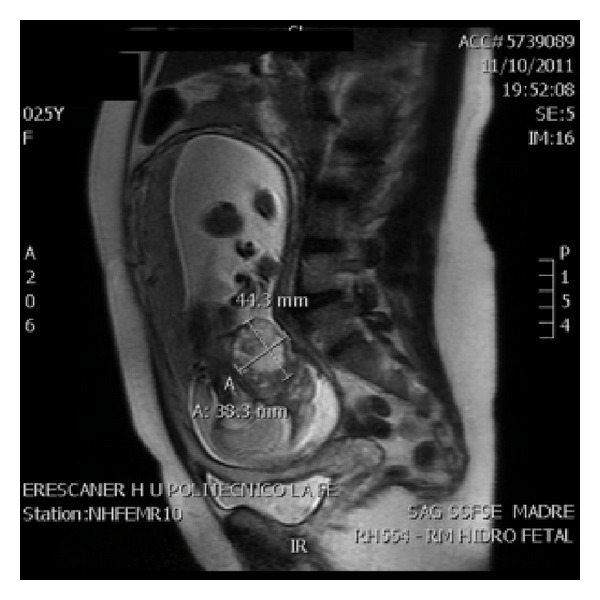
Nuclear magnetic resonance at 25 weeks, showing heterogeneous mass in cervical situation.

A primigravida, 25-years-old patient, it showed a cervical tumor heterogeneous solid-liquid content of 43 × 44 mm, moderately vascularized, in ultrasound study in week 22 (Figure 1), with male fetus and normal anatomical exploration, visualizing gastric chamber. We propose to make a genetic amniocentesis, but the patient rejected it. Magnetic resonance (MR) was requested in the 25th week of gestation, describing 56 × 43 × 38 mm heterogeneous mass in anterior cervical position, with high bilateral extension, solid-cystic component, without thoracic infiltration (Figure 2). We suspect a congenital cervical teratoma by ultrasound and MR images.

**Figure 3:**
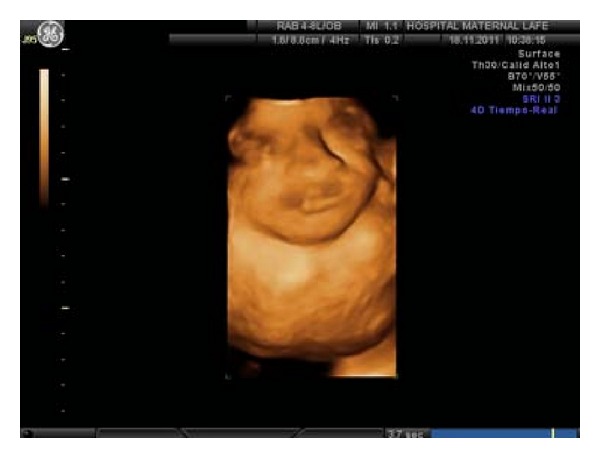
3D ultrasound.

New ultrasound study in 26 +5 weeks, shows a significant growth of the mass, 69 × 53 mm size (Figure 3). The patient wishes to continue the pregnancy. New MR control in week 29, shows an increase of significant size, high anterior growth, without existing intrathoracic extension. Similar proportion of solid and cystic component, with a sagittal plane measurement of 61 × 63 mm and coronal plane 51 × 93 mm, with partial compression of the air-digestive track, but an adequate fetal stomach fullness.

**Figure 4:**
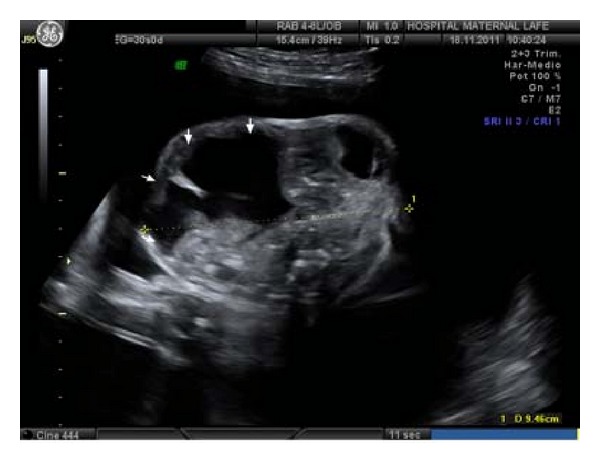
New ultrasound control in week 30, which maintains the increased tumor size (arrows) and heterogeneous component.

During follow-up controls, we observe in 30th week, a 94 × 65 × 61 mm size with similar characteristics previously described, but polyhydramnios apparition, with stomach well visualized. Probably secondary to the large mass compressive effect on the air-digestive track (Figure 4).

With all these findings, we explain the fetal management in the delivery moment to the couple, and they understood and accepted the maternal and fetal risks. It is so important with this pathology, the prevention of acute respiratory failure (asphyxia) after the birth, which would result in the death of the baby if there is not an active management, so we propose EXIT surgery (ex utero intrapartum treatment).

So we create a multidisciplinary EXIT team, to be able to make a safe surgery management. It was necessary the participation of Obstetrics, Neonatology, Children's and Adult Anesthesia, Children's Otolaryngology and Pediatric Surgery. After the case study, it is decided to finish pregnancy at 35 gestational week, with previous fetal lung maturation.

**Figure 5:**
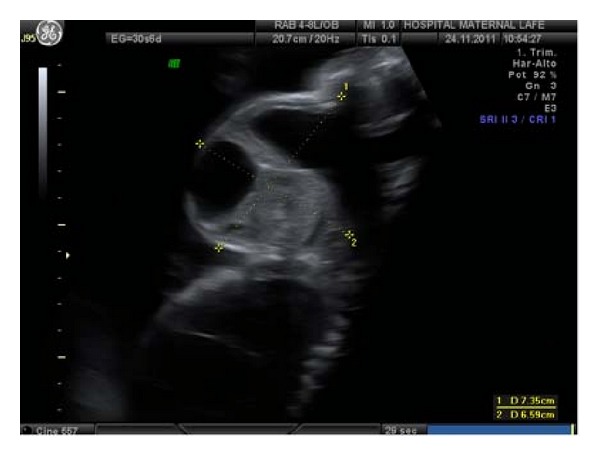
Lateral view of the last ultrasound control before surgery.

The patient performs weekly ultrasound controls until the surgery date. She was hospitalized from the polyhydramnios appearance. At 31 +4 weeks, an 1518 grams fetal weight estimated, with a 95 × 70 × 100 mm tumor size. All time the fetus has biometry and UA Doppler normal for gestational weeks (Figure 5).

In the 31 +6 week, the patient begins with uterine dynamics, the ultrasound examination shows a severe polyhydramnios with cervical change, with a cervical length of 0.5 cm, with funneling of 0.85 cm. So we decided to make an amniodrainage, which removed approximately 3000 mL of clear amniotic fluid, with a 0.91 cm cervical length after that. The patient went into labor the same day, so EXIT was performed at 32 gestational weeks.

**Figure 6:**
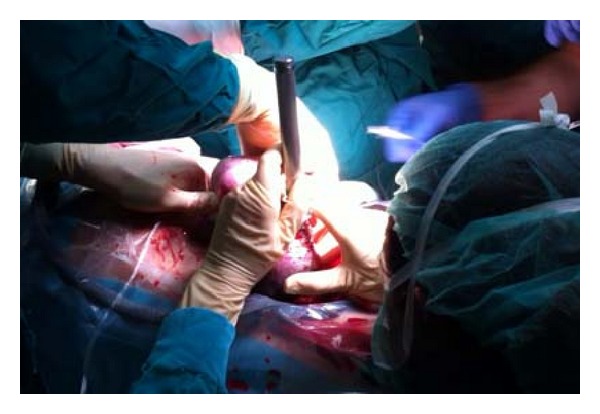
Fetal intubation time during EXIT.

After general anesthesia with endotracheal intubation and constant patient monitoring, we made an hysterotomy to extract in 7 minutes the head and fetal neck, maintaining uteroplacental circulation, we could see a large tumor covering all anterior fetal neck. At the same time, neonatal intubation in was done situ in three tries. After verifying the air safety, extraction of total body and cord clamping it was carried out. After this, we proceeded extraction placenta, uterine integrity check, and hysterorrhaphy, verifying good uterine contraction. Surgery duration was 95 minutes (Figure 6).

The patient had a normal postoperative period, with early discharge two days later.

The neonate was admitted immediately in neonatal ICU. One day after cesarean procedure, he was operated by pediatric surgery, an was observed exophytic cervical mass approx. 10 × 15 cm, with soft consistency and vascularized solid-cystic component, as described by the ultrasound study. They had a good cleavage plane, and they could resect totally with 160 grams surgical piece weight. The mass extended up sides tracheal and sternal notch, it did not infiltrating thyroid gland.

The pathology study gave the definitive diagnosis, being an immature cystic teratoma grade II (high grade). The microscopy study showed abundant growth from different tissues (cartilage, colon, bone, choroid plexus, retina, etc.)

Nowadays, one year after surgery, the neonate is controlled by the pediatric oncology with monthly AFP determination with serial cervical ultrasound studies for assessing the presence of remaining tumor. He is currently asymptomatic, with a growth and development correct.

## 3. Discussion

The presence of pathology in the cervical position generates an important risk to the extrauterine viability, it forces to make an aggressive management of this pathology, as the case presented.

The presence of cervical teratomas, constitutes about 3% of neonatal teratomas, with low frequency, but they can generate significant neonatal morbidity and mortality because of the location and management [1, 2].

The natural history of congenital obstruction of the airway and digestive track is not well known. We know that the blockade creates a narrowing, so that it reduces the swallowing fetal amniotic fluid into stomach and it generates a dilatation of the preblock structures, and it adds difficulties of respiratory fluid excretion to amniotic cavity, so it explains the appearance of polyhydramnios [3, 4]. It is very important in all ultrasound examinations to check the correct filling fetal stomach.

This pathology requires an adequate ultrasonic early diagnosis and followup. We can extend our study with the use of other imaging techniques such as MRI, it helps especially in the differential diagnosis of a cervical tumor in the fetal study, in our case, thyroid origin was rejected because it had not the central position of the gland and the typical image of hyperintensity on T1 MRI. At the same time, lymphangioma was rejected by excessive present solid component, it would orient more a venolymphatic malformation. 

The EXIT procedure (ex utero intrapartum treatment) is based on a multidisciplinary approach, looking for maintaining airway patency in extrauterine life. It uses the nasotracheal intubation, laryngeal mask, or tracheostomy, while uteroplacental circulation is maintained. So in this way, you can avoid the asphyxia due to tumor compressive effect, and in a second time, you will treat the obstruction.

The EXIT surgery gives to you a 45–60 minutes working time, to ensure the control of the neonatal airway, while the fetus has uteroplacental perfusion [5]. In our case, the intubation was difficult, but we were able to avoid the need for tracheostomy, with neonatal airway control in 4 minutes.

This technique involves many risks. You must maintain an adequate uteroplacental perfusion with a good uterine relaxation, a mean arterial pressure above 65 mmHg with CVP around 10 cmH20 is recommended [6]. So, you get an excellent fetal blood perfusion, but it is frequent with a poor uterine relaxation, the appearance of arterial desaturation, bradycardia, placental abruption, or umbilical cord compression. If you have a decrease in arterial perfusion, you will have fetal brain injury, secondary to hypoxia.

The most important maternal complications are the bleeding, you have so much time an intense uterine musculature relaxation to maintain a correct fetal perfusion, so the bleeding is higher. You must maintain the relaxation with the use of short half-life drugs, in order to delete quickly their effects after delivery. And the intensive use of Oxytocin, Ergometrine, or Carbetocin to get a well contracted uterus after the EXIT procedure is important. Another complication is the risk of endometritis by the manipulation that you have in the surgical place, so the use of prophylactic antibiotics is required.

With all this information, the surgical complication rate is low. Hedrick et al. published a review of 42 EXIT cases, where they had only two puerperal endometritis and the need of blood transfusion in two patients [7].

## 4. Conclusions

The EXIT approach is an appropriate tool in the congenital airway obstruction management, because it allows the fetal adaptation into extrauterine life.

This procedure requires a multidisciplinary participation, it involves many professionals from different specialties, with a preoperative detailed planning. The detailed followup during the pregnancy is very important, you must be always conscious of the maternal-fetal wellbeing during the process.

At the same time, the ultrasound and MRI evaluation give to you the diagnosis and management option of congenital cervical pathology, as the case presented. Our therapeutic decision was based on the information obtained by ultrasonic monitoring.
